# The effect of immune cell‐derived exosomes in the cardiac tissue repair after myocardial infarction: Molecular mechanisms and pre‐clinical evidence

**DOI:** 10.1111/jcmm.16686

**Published:** 2021-06-05

**Authors:** Heling Wen, Lei Peng, Yu Chen

**Affiliations:** ^1^ Department of Cardiology Sichuan Academy of Medical Science & Sichuan Provincial People's Hospital Chengdu China; ^2^ Department of Nephrology Sichuan Academy of Medical Science & Sichuan Provincial People's Hospital Chengdu China

**Keywords:** cardiomyocyte, exosome, immune cell, inflammation, myocardial infarction

## Abstract

After a myocardial infarction (MI), the inflammatory responses are induced and assist to repair ischaemic injury and restore tissue integrity, but excessive inflammatory processes promote abnormal cardiac remodelling and progress towards heart failure. Thus, a timely resolution of inflammation and a firmly regulated balance between regulatory and inflammatory mechanisms can be helpful. Molecular‐ and cellular‐based approaches modulating immune response post‐MI have emerged as a promising therapeutic strategy. Exosomes are essential mediators of cell‐to‐cell communications, which are effective in modulating immune responses and immune cells following MI, improving the repair process of infarcted myocardium and maintaining ventricular function via the crosstalk among immune cells or between immune cells and myocardial cells. The present review aimed to seek the role of immune cell‐secreted exosomes in infarcted myocardium post‐MI, together with mechanisms behind their repairing impact on the damaged myocardium. The exosomes we focus on are secreted by classic immune cells including macrophages, dendritic cells, regulatory T cells and CD4^+^ T cells; however, further research is demanded to determine the role of exosomes secreted by other immune cells, such as B cells, neutrophils and mast cells, in infarcted myocardium after MI. This knowledge can assist in the development of future therapeutic strategies, which may benefit MI patients.

## 1 INFLAMMATION PROCESS AND INFARCTED MYOCARDIUM

Myocardial infarction (MI), representing a major cause of mortality and morbidity in cardiovascular disease worldwide, occurs when blood flow to the myocardium is suddenly blocked by a partial or complete blockage of a coronary artery, leading to cardiomyocyte damage and ischaemia.^1^, ^2^ The cardiac damage after MI is a potent trigger to activate the immune responses, which assists to repair ischaemic injury and restore tissue integrity through the modulating different steps of healing process after MI. Acute cardiac injury leads to local inflammation, activation of endothelial cells in the vasculature and overexpression of adhesion molecules like integrins to recruit immune cells. Indeed, injured cardiomyocytes post‐MI undertake the necrotic process and release the danger‐associated molecular patterns (DAMPs) into the extracellular environment, which promote an acute inflammatory response to digest and clear necrotic debris and extracellular matrix (ECM) tissue.^3^ DAMPs, such as heat shock proteins like high mobility group box 1, induce responses of the immune system via binding to cognate pattern recognition receptors, including nucleotide‐binding oligomerization domain‐like receptors and toll‐like receptor/interleukin 1 receptors (TLR/IL1R), on living cardiomyocytes.^4^, ^5^, ^6^, ^7^ Activated receptors promote the secretion of different pro‐inflammatory mediators and thus lead to the intercellular crosstalk signal. Cardiomyocyte‐secreted chemokines bind to the related receptors and trigger extravasation and recruitment of immune cells. Increased production of pro‐inflammatory cytokines, including tumour necrosis factor (TNF), interleukin 6 (IL‐6) and interleukin 1β (IL‐1 β), induces adhesive interactions between recruited immune cells and endothelial cells. Taken together activation of chemokines and inflammatory cytokines causes transmigration of a huge number of inflammatory cells into infarcted myocardium.^8^

The transient and intense MI‐promoted inflammatory responses play critical roles in cardiac repair and clearing the infarcted area of injured and dead cells and ECM debris.^9^ After MI, the inflammatory process occurs during two different temporal phases, including an early inflammatory phase followed by a reparative phase.^9^ Neutrophils are the first immune cells recruited to the myocardium. These immune cells induce macrophage infiltration and have critical role in the cardiac repair and survival.^9^ In the early phase of inflammatory process immediately after MI, the pro‐inflammatory chemokines and cytokines are secreted from M1 macrophages to provoke inflammatory responses to degrade and remove necrotic or injured cells. Through the following days, the inflammatory phase gently shifts to the reparative phase involving inflammation resolution, neovascularization and scar generation. The switching to the reparative stage needs the in time inhibition of the inflammatory process through the activity of immune‐suppressive lymphocytes and anti‐inflammatory M2 macrophages.^9^ During the reparative phase, cardiac macrophages polarize to M2 phenotype secreting pro‐fibrotic and anti‐inflammatory cytokines like IL‐10 and transforming growth factor‐beta (TGF‐β), which inhibit inflammation and induce tissue repair. M2 macrophages also trigger ECM generation and angiogenesis via secreting the vascular endothelial growth factor (VEGF) and TGF‐β.^10^ In addition, bone marrow‐derived dendritic cells infiltrate the necrotic regions of the myocardium, mainly through the reparative phase.^11^, ^12^ Infiltrated dendritic cells (DCs) seem to regulate macrophage homeostasis after MI, therefore, modulating the post‐infarction healing process.^12^ Besides, T cells are also found to involve in the inflammatory process to varying degrees in the infracted myocardium after MI.^13^

Although the inflammatory process leads to clearing damaged myocardial cells and facilitating scar formation, an inefficient early immune response can cause the fatal cardiac rupture while excessive or prolonged inflammation response promotes the ECM degradation and the adverse cardiac remodelling causing heart failure (HF).^14^ Of note, a regulated balance between the promotion and suppression of inflammatory responses post‐MI provides a subsequent proliferative phase of healing in the infarcted area. Thus, a timely resolution of inflammation and a firmly regulated balance between regulatory and inflammatory mechanisms is critical. Immune cells, rather than damaged cardiomyocytes, are the principal modulators of such balance. Therefore, modulating inflammation response after MI may provide a potential approach to diminish myocardial dysfunction. Notably, there is growing evidence showing the essential role of immune cell‐derived exosomes in these functions,^15^, ^16^, ^17^, ^18^ and thus, in the present review, we aimed to summarize the immunomodulatory impacts of immune cell‐secreted exosomes on cardiac repair after MI.

## 2 EXOSOMES

A vital route of intercellular communication is provided by exosomes that are released by cells to extracellular space and participate in various physiological processes such as immune regulation.^19^ Exosomes are nano‐sized (30‐120 nm) lipid bilayer extracellular vesicles that are secreted by almost all cell types and carry a wide range of biologically active cargos, including cell‐specific proteins, lipids and genetic materials like mRNAs and microRNAs (miRs), to the target cells.^20^ Exosomes can target specific cell types, depending on their origin cell, molecular contents and surface antigens. By transporting biologically active cellular constituents from the donor to the recipient cells, exosomes modulate the function and behaviour of recipient cells under both normal and pathological conditions.^21^, ^22^ Exosomes participate in cell‐to‐cell communication through various routes, including receptor/ligand interaction in which exosomal transmembrane proteins bind to the signalling receptors of recipient cells^23^; fusion with the plasma membrane of recipient cells and deliver their contents into cytosol; or cellular internalization via caveolin‐ or clathrin‐dependent endocytosis^24^, ^25^ as well as cellular uptake through phagocytosis or micropinocytosis.^26^, ^27^

With exosomes being growingly studied and used to cardiovascular disorders like MI,^28^ it is expectable that, considering well‐established cell‐based immunotherapies, immune cell‐derived exosomes will provide new effectual substitutes for patients. Exosome‐based cell‐free therapeutic approaches that recapitulate and even intensify the impacts of cell therapies show numerous advantages. The main concerns regarding cell therapy, which can be bypassed via exosomes, are the possibility for incidence of cellular embolism following intravenous injection, hardness of passing through the blood‐brain barrier, ectopic tissue formation, infusion‐related toxicity resulted from transplanted cells embedded in the pulmonary microvasculature, the low cell viability, the immune rejection and tumorigenicity, as well as safety and ethical issues.^29^, ^30^, ^31^, ^32^ Regarding the handling and manufacturing processes, exosome‐based therapeutic approaches demand simple preparation and sterilization steps and require easier storage conditions,^28^ considerably decreasing the total cost in comparison with cell‐based therapies. Additionally, the general stability and safety of exosomes secreted by various cell types have been approved in a number of clinical trials (50). However, up to now, there have been no clinical trials evaluating exosomes for treating cardiovascular disease, and thus, the safety and practicability of exosome‐based therapeutic approaches in this field remain to be approved.

The present paper would review the emerging role of immune cell‐derived exosomes in infarcted myocardium after MI, together with mechanisms underlying their repairing effects on the injured myocardium (Table 1). The exosomes we focus on are secreted by classic immune cells including macrophages, dendritic cells, regulatory T cells (Tregs) and CD4^+^ T cells; however, further research is demanded to determine the role of exosomes secreted by other immune cells, such as B cells, neutrophils and mast cells, in infarcted myocardium after MI. This knowledge can assist in the development of future therapeutic strategies, which may benefit MI patients.

**TABLE 1:** Molecular targets and mechanisms underlying impacts of exosomal miRs secreting by various immune cells after MI

Immune cells	Exosomal miRs	Target cells	Molecular targets	Physiological effects after MI	Underlying mechanisms	Ref.
Dendritic cell	miR‑494‑3p	CMECs	VEGF	‐ Enhancing angiogenesis ‐ Ameliorating the infarcted myocardium	‐	52
Macrophage	miR‐155	Cardiac fibroblasts	‐ SOCS1 ‐ SoS1	‐ Intensifying cardiac inflammation ‐ Inducing cardiac rupture	‐ Suppressing fibroblast proliferation ‐ Increasing the expression of TNF‐α, IL1β, and CCL2 ‐ Reducing the production of collagen	80
M1 macrophage	miR‐155	CMECs	‐ RAC1 ‐ PAK2 ‐ Sirt1 ‐ AMPKα2	‐ Suppressing angiogenesis ‐ Exacerbating myocardial injury ‐ Hindering cardiac healing	‐ Depressing Sirt1/AMPKα2‐endothelial nitric oxide synthase ‐ Depressing RAC1‐PAK2 signalling pathways	82
M2 macrophage	miR‐148a	Cardiomyocytes	TXNIP	‐ Increasing the viability of injured cardiomyocytes ‐ Reducing the infarct size ‐ Ameliorating MI/R injury	‐ Inactivating the TLR4/NF‐κB/NLRP3 signalling pathway ‐ Alleviating Ca^2+^ overload ‐Regulating cardiac enzymes like CK, CK‐MB, and lactate dehydrogenase	75
CD4^+^ T cells	miR‐142‐3p	Cardiac fibroblasts	APC	‐ Triggering myofibroblast activation and fibrogenesis ‐ Deteriorating cardiac fibrosis ‐ Worsening cardiac dysfunction ‐ Inducing abnormal cardiac remodelling	‐ Inducing β‐catenin degradation ‐ Modulating GSK‐β‐β‐catenin signal cascade ‐ Increasing TGF production	116

Abbreviations: AMPKα2, AMPactivated catalytic subunit alpha 2; APC, Adenomatous Polyposis Coli; CCL2, C‐C Motif Chemokine Ligand 2; CK, Creatine Kinase; CK‐MB, Creatine Kinase Myocardial Band; CMECs, Cardiac Microvascular Endothelial Cells; GSK‐β, Glycogen Synthase Kinase beta; IL‐1β, Interleukin 1 beta; MI, Myocardial Infarction; miRs, microRNAs, NF‐κB, Nuclear factor‐κB; NLRP3, NLR family Pyrin domain containing 3; PAK2, p21 (RAC1)‐Activated Kinase 2; RAC1, Rac family small GTPase 1; Sirt1, Sirtuin 1; SOCS1, Suppressor of Cytokine Signalling 1; SoS1, Son of Sevenless gene 1; TGF, Tumour Growth Factor; TNF‐α, Tumour Necrosis Factor‐alpha; TXNIP, Thioredoxin‐Interacting Protein; VEGF, Vascular Endothelial Growth Factor.

### 2.1 The role of dendritic cells in post‐myocardial infarction

DCs are the most efficient member of the professional antigen‐presenting cells (APCs) that play as the key and impressive immunoregulators modulating various lines of immune cells in innate and adaptive immunity.^33^ Emerging data indicate that DCs have critical roles in the pathophysiological mechanisms of various cardiovascular disorders, especially MI.^34^ In the infarcted myocardium, DCs are essential for the recruitment and activation of immune cells, especially T cells and macrophages, accompanied by a marked elevation of inflammatory cytokines.^35^ Notably, it has been recently found that the infiltration of DCs is remarkably increased in the infarcted myocardium and, in turn, the frequency of circulating DCs is decreased in post‐MI.^11^, ^12^, ^36^ After MI, DCs migrate to the watershed infarcts and participate in the activation of lymphocytes and the promotion of immune responses.^35^, ^37^ In vivo studies have shown that DC ablation in mice leads to the increased and sustained production of inflammatory cytokines like TNF‐α, IL1β and IL18, prolonged degradation of the extracellular matrix (ECM), and increased recruitment of pro‐inflammatory M1 macrophages in post‐MI.^12^ Of note, treatment of the infarcted mice with DCs accelerated macrophage polarization to M2 subtype and promoted a systemic activation of MI‐specific Tregs, causing improved wound healing and preserved systolic activity of left ventricular.^38^

#### 2.1.1 Dendritic cell‐secreted exosomes

DCs secrete EXs that mediate intercellular communication in the immune system. DC‐secreted EXs (DC‐EXs) show non‐negligible similarity to the parent DCs in terms of membrane proteins and biological functions. DC‐EXs express immune‐stimulatory molecules, deliver antigen‐specific signals and are known as inert carriers that recapitulate functions of DCs and target and activate other immune cells.^39^, ^40^, ^41^, ^42^, ^43^ Similar to DCs, DC‐EXs are molecularly composed of the functional complexes of surface MHC‐peptides, T cell co‐stimulatory molecules and other molecular elements required for interacting with immune cells.^44^ The CC‐chemokine receptor 7 (CCR7), which guides mature DCs to peripheral lymphoid organs such as the spleen, was shown to comparably regulate inflammatory responses and the elevated accumulation of EXs secreted by mature DCs in the spleen after administration to mice.^45^ DC‐EXs can also do antigen presentation either by self or by snatching the plasma membrane of parent DCs through their release.^33^ Although the biological functions of DC‐EXs have been studied mainly in cancer cells,^40^, ^41^, ^46^, ^47^, ^48^ growing attention has recently been attracted to the role of DC‐EXs in MI.

#### 2.1.2 The role of dendritic cell‐secreted exosomes in post‐myocardial infarction

Mounting data suggest that DC‐EXs have advantages over cardioprotective immunotherapies exploiting DCs. After MI in mice, it was shown that DC infiltration is markedly elevated in the infarcted area^11^, ^49^, ^50^ and the percentage of mature DCs secreting EXs is significantly increased among cardiac DCs, which is attributed to the post‐MI microenvironment of injured cardiomyocytes.^51^ Such MI‐DC‐EXs could directly activate splenic CD4^+^ cells.^51^ MI‐DC‐EXs were found to induce significant up‐regulation of the chemokines MIP‐1 and MCP‐1 in CD4^+^ cells, which led to greater and more rapid migration and recruitment of additional DC‐EXs into the spleen.^51^ Activated CD4^+^ T cells have been found to play a determinant role in improving myocardial wound healing post‐MI.^13^ Notably, injected splenic CD4^+^ T cells activated by MI‐DC‐EXs or activated CD4^+^ T cells that had uptaken DC‐EXs could deliver into the infarcted myocardium and eventually improve the cardiac function of mice post‐MI.^51^ As the number of anti‐inflammatory CD4^+^ Foxp3^+^ Tregs between CD4^+^ T cell population in heart‐draining lymph nodes is known to be elevated in the wake of MI,^51^ it can be postulated that DC‐EXs likely activate cardioprotective Tregs after infarction and, thereby, beneficially impact wound healing and survival.

Besides, there is evidence showing the key role of DC‐EXs in angiogenesis following MI.^52^ During the early phase after MI, angiogenesis plays an important role in the recovery of the injured myocardium and myocardial remodelling as well as maintenance of cardiac function^53^, ^54^ through healing the microvascular bed and preventing further necrosis and apoptosis of ischaemic myocardial tissue, which may be damaged, hibernating or stunned following MI.^53^ Notably, DC ablation has been found to deteriorate angiogenesis and cardiac function in mice post‑MI,^12^ supporting the determinant role of DCs in angiogenesis and cardiac healing after MI. Of note, DC‐EXs are one of the critical factors that mediate such an effect. Recently, it has been shown that the DCs infiltrating the infarcted myocardium following MI could induce angiogenesis by secreting the EXs containing highly expressed angiogenic miRs, particularly miR‑494‑3p.^52^ It was found that miR‑494‑3‐enriched MI‐DC‐EXs could be directly taken up by the cardiac microvascular endothelial cells (CMECs), significantly up‐regulate the expression of the VEGF in CMECs, and thereby enhance angiogenesis in the infarcted myocardium after experimental MI.^52^

In conclusion, MI‐DC‐EXs can promote recovery of the injured myocardium and improve cardiac function after MI through activating CD4^+^ Foxp3^+^ Tregs cells and inducing angiogenesis, providing a potent strategy for the treatment of MI.

### 2.2 The role of macrophages in post‐myocardial infarction

Macrophages are a group of heterogeneous monocyte/macrophage lineage cells due to the plasticity of the cells in the various milieu. Macrophages display diverse phenotypes in response to different stimuli, and they are thus classified into two subsets, including classically activated macrophages (M1) produce pro‐inflammatory cytokines^55^ and alternatively polarized M2 macrophages suppressing immune responses, supporting Th2 immunity, and promoting tissue regeneration and remodelling as well as wound healing.^56^

Macrophage modulation has been known to provide a vital regulator upon cardiac remodelling and repair after MI. Following MI, cardiomyocyte death promotes an inflammatory reaction to digest and eliminate injured cells and extracellular materials. Macrophages act as dominant immune cells that regulate the inflammation progression and resolution.^57^, ^58^ Several hours post‐MI, during healing and remodelling after cardiac injury, monocytes spread into the infarcted myocardium and then gradually polarize to macrophages in the microenvironment of MI.^59^, ^60^ M1 and M2 phenotypes show distinguished functions and consequently resolve or exacerbate inflammation in the infarcted myocardium. During the inflammatory phase after MI, M1 macrophages, which are highly phagocytic and secrete massive levels of pro‐inflammatory cytokines such as TNF‐α and IL‐1β, and proteinases, such as matrix metalloproteinases (MMPs), are remarkably increased and aid in clearing cellular debris.^61^, ^62^ Afterwards, during the reparative phase post‐MI, macrophages in the infarcted myocardium dominantly shift from M1 into M2 phenotype producing anti‐inflammatory/reparative cytokines, such as TGF‐β, VEGF and IL‐10, which tune the anti‐inflammatory and reparative response and facilitate wound repair by activation of myofibroblasts, angiogenesis and the ECM deposition.^62^, ^63^, ^64^, ^65^

Though macrophages with M1 phenotype exert a positive effect, their long‐time presence prolongs the pro‐inflammatory step and accelerates cellular death and ECM degradation. Such an event leads to the enlargement of the infarcted region and unintended hindrance of cardiac repair following MI.^66^, ^67^, ^68^ The exceeding pro‐inflammatory function of M1 macrophages induces cardiomyocyte death,^66^ pervades fibrosis,^69^ inhibits neovascularization^70^ and hamper myocardial regeneration,^68^ resulting in the high risk of cardiac rupture.^71^ Therefore, a regulated balance between M1 and M2 phenotypes of macrophages is essential for cardiac repair. Following MI, an in time inflammation resolution via the impact of M2 macrophages is necessary for appropriate myocardial repair and suppression of abnormal remodelling. On the other hand, targeting M1 macrophages, which reduces the duration of the pro‐inflammatory state, can enhance the functionality of cardiac tissue post‐MI.^72^ Thus, inducing the M1/M2 shift or dynamic balance has received an attractive concept of a new target for MI therapy.^64^, ^65^, ^73^

Macrophages can also abundantly secrete EXs that show both pro‐ and anti‐inflammatory activity in diverse contexts, partly because of previously activated macrophages that have already been primed with stimuli. There have been investigations comparing EXs secreted from M1 (M1‐EXs) and M2 (M2‐EXs) macrophages.^74^, ^75^ There is evidence that shows, similar to their parent cells, M2‐EXs exhibit contrary functions to M1‐Exos in the tumour biology^76^, ^77^ and MI, as discussed in the following sections.

#### 2.2.1 The role of M1‐EXs in post‐myocardial infarction

Macrophages through the crosstalks with cardiac fibroblasts induce cardiac remodelling after MI.^78^, ^79^ Of note, EXs secreted from infiltrated macrophages (Mac‐EXs) were shown to impact the function of fibroblasts. In the mouse heart post‐MI, it was found that cardiac Mac‐EXs containing miR‐155 could move into cardiac fibroblasts and mediate macrophage‐fibroblast crosstalk.^80^ It can be further supported by a study that showed MI significantly elevates the levels of mir‐155 in cardiac fibroblasts.^81^ Notably, it was shown that mir‐155 expression is highly up‐regulated in cardiac cells in response to angiotensin II, which can result in the active packaging of mir‐155 into cardiac Mac‐EXs that are taken up by fibroblasts.^80^ In mechanism, angiotensin II‐stimulated macrophages were found to secrete mir‐155‐enriched EXs that could inhibit proliferation of cardiac fibroblasts by down‐regulating the expression of Son of Sevenless gene 1 (SoS1) and induce cardiac inflammation accompanied by increasing the expression of C‐C Motif Chemokine Ligand 2 (CCL2), TNF‐α and IL1β by inhibiting the expression of suppressor of cytokine signalling 1 (SOCS1).^80^ Mac‐EXs containing miR‐155 could also reduce the collagen generation and induce cardiac rupture, which is mediated by SOCS1 and SoS1 targeting.^81^ Interestingly, such effects were reversed in miR‐155‐deficient mice, which showed a marked decrease in the incidence of cardiac rupture and an enhanced cardiac function, elucidating the critical role of cardiac Mac‐EX‐mir‐155.^80^ Further study indicated that miR‐155‐enriched EXs are also highly secreted from pro‐inflammatory M1 macrophages after MI and exert an anti‐angiogenic impact and accelerate MI damage.^82^ Similar to the just‐mentioned Mac‐EXs, EXs secreted by M1 macrophages (M1‐EXs) deliver pro‐inflammatory miR‐155 to endothelial cells, resulting in the angiogenesis suppression and cardiac dysfunction via down‐regulation of relevant genes, such as protein kinase AMP‐activated catalytic subunit alpha 2 (AMPKα2), Sirtuin 1 (Sirt1), Rac family small GTPase 1 (RAC1) and p21 (RAC1)‐activated kinase 2 (PAK2).

M1‐EXs inhibit Sirt1/AMPKα2‐endothelial nitric oxide synthase and RAC1‐PAK2 signalling pathways via simultaneous targeting the just‐mentioned molecule nodes, decrease the angiogenic ability of endothelial cells, exacerbated myocardial injury and hinder cardiac healing.^82^ Interestingly, macrophages themselves have been also found to be recipients of miR‐155‐enriched EXs from endothelial cells, which further orientates the polarization of macrophages from anti‐inflammatory M2 phenotype to pro‐inflammatory M1 phenotype.^83^

Besides EX‐miR‐155, increased levels of many other pro‐inflammatory miRs, such as miR‐223, miR‐146, miR‐21 and miR‐19, have been also detected in the plasma circulating EXs (with monocytic origin) from patients with the acute coronary syndrome (ACS), when compared with patients with stable coronary artery disease (CAD),^84^ which can donate visions for detecting other important miRs in M1‐EXs.

#### 2.2.2 The role of M2‐EXs in post‐myocardial infarction

The MI, due to occlusion of coronary arteries, results in transmural myocardial ischaemia that, in turn, leads to irreversible death of cardiomyocytes and myocardial injury or necrosis.

Though in time and absolute myocardial reperfusion is the most effectual approach to protect the myocardium after MI, restoration of the coronary blood urges further irreversible damage to the myocardium and it leads to final infarct size (INF), termed as ‘myocardial ischemia/reperfusion’ (MI/R) injury.^85^ The pathophysiology of MI/R injury is not precisely known. A reduced potential of inner mitochondrial membrane and elevated levels of reactive oxygen species (ROS), inorganic phosphate or calcium ions is found in MI/R injury, which may result in mitochondrial permeability transition pore (MPTP) and further increase cell apoptosis.^86^ Although using the antithrombotic and antiplatelet drugs are the standard strategy to preserve the patency of the infarct‐related coronary artery, there is no therapeutic approach to sufficiently protect against MI/R injury, while the life‐threatening reperfusion attributes to more than 50% of the final INF.^87^ Therefore, developing novel therapeutics better able to decrease infarct size represents a major challenge.^86^

Switching harmful and inflammatory M1 macrophages towards anti‐inflammatory M2 macrophages with ameliorating effects is found to protect against various kinds of I/R injuries.^88^, ^89^, ^90^ Emerging studies suggest that exosomes may serve as key mediators in MI/R injury. It has been recently shown that M2‐EXs can protect MI/R injury.^75^ M2‐EXs were found to transport high levels of miR‐148a into cardiomyocytes and, consequently, increase the viability of injured cardiomyocytes and alleviate Ca^2+^ overload and dysregulation of cardiac enzymes known as MI biomarkers like creatine kinase, creatine kinase myocardial band (CK‐MB) and lactate dehydrogenase, leading to a reduction in infarct size and MI/R injury after experimental MI.^75^ Such effects are mediated by down‐regulating the thioredoxin‐interacting protein (TXNIP) and inactivating the TLR4/NF‐κB/NLRP3 inflammasome signalling pathway.^75^ Mechanistically, miR‐148 can reduce the TXNIP via directly binding to its 3' untranslated region (3'UTR).^91^ TXNIP plays the important role in redox homeostasis and has increased levels in MI/R, whose overexpression can cause cellular apoptosis by elevating levels of ROS and oxidative stress.^92^ TXNIP has been documented to closely link to the phosphorylation and activation of TLR4/NF‐κB/NLRP3 inflammasome pathway^93^, ^94^, ^95^ that is correlated with various cardiovascular diseases.^96^ Furthermore, there is evidence that shows M2‐EXs could modulate the signalling of various biomolecules from M1‐EXs, thereby inhibiting the inflammatory function of M1 macrophages in post‐MI. For instance, the expression level of pro‐inflammatory miR‐155, which is highly expressed in M1 macrophages (as discussed in the last section), is lower in M2 phenotype than in M1,^97^, ^98^ and it has been found that M2‐EXs cytokines, such as VEGF, reduce the expression levels of cellular and exosomal miR‐155 in macrophages.^82^

### 2.3 The role of Treg‐secreted exosomes in post‐myocardial infarction

The maintenance of cardiac function after the MI needs structural repair. Myocardial repair is a complicated process relying on the activation of inflammatory response that acts as a double‐edged sword. On the one hand, uncontrolled inflammatory response causes an enlargement in the size of myocardial infarct size and deteriorates reverse cardiac remodelling. On the other hand, insufficient inflammatory response influences macrophage‐mediated phagocytosis of necrotic debris, which in turn impacts myocardial repair.^10^ Indeed, the cardiac repair response can be promoted only after subsiding the inflammatory response. Tregs, as a particular subtype of CD4^+^ lymphocytes with immunosuppressive impacts, have a fundamental role in promoting the polarization of anti‐inflammatory M2 macrophages, increasing the production of anti‐inflammatory cytokines such as IL‐10, IL‐4 and IL‐13, and decreasing the levels of pro‐inflammatory cytokines.^99^, ^100^ There is evidence showing the crucial role of Tregs in MI and cardiac remodelling after MI. Of note, the circulating levels of Tregs are markedly reduced in patients with chronic heart failure, and their anti‐inflammatory functions are notably compromised in such patients.^101^ Animal studies indicated large infarct size and poor survival rates in Treg‐depleted mice and, in contrast, few ventricular ruptures, negligible levels of heart failure and the long survival time in transgenic mice with the high frequency of Tregs in the infarct site.^100^, ^102^ Notably, the Treg injection was found to decrease infarct size and improve MI.^38^ Such findings may be attributed to the events in which Tregs maintain the stability and dynamic balance of the internal environment. Following MI, Tregs infiltrate into the myocardium and induce the differentiation of M2 macrophages and highly secrete anti‐inflammatory factors, like IL‐10 and TGF‐β, to inhibit the inflammatory response of M1 macrophages and lymphocytes within the healing myocardium, thereby ameliorating the inflammation‐mediated cardiac damage and improving wound healing.^100^, ^103^ They also decrease excessive ECM degradation and regulate the phenotype and function of fibroblasts, thus alleviate myocardial fibrosis and cardiac remodelling after MI.^100^, ^103^, ^104^

Exosomes have been found to precipitate in the immunosuppressive function of Tregs through inhibiting the pro‐inflammatory function of effector T cells^105^, ^106^, ^107^ and inducing a tolerogenic phenotype in DCs producing less pro‐inflammatory cytokine IL‐6 and more anti‐inflammatory cytokine IL‐10.^108^ Treg‐secreted exosomes (Treg‐EXs) were found to markedly decrease the myocardial infarct size and suppress apoptosis of myocardial cells in mice with MI, which was along with a reduction in the M1 marker iNOS and an increase in Arg‐1 M2 marker as well as a reduction in the pro‐inflammatory mediators, such as IL‐1β and TNF‐α, and an increase in the anti‐inflammatory mediators, such as IL‐10 and TGF‐β.^109^ As anti‐inflammatory M2 macrophages play crucial roles in the wound healing response and the fibrovascular scar formation,^110^ these findings imply that Treg‐EXs could ameliorate the MI by inducing macrophage polarization towards the M2 phenotype. To sum up, Treg‐EXs can exert a cardioprotective effect after MI by interacting with cardiac macrophages.

### 2.4 The role of CD4^+^ T cell‐secreted exosomes in post‐myocardial infarction

Although the sterile inflammation promoted by necrotic cardiomyocytes activates post‐MI cardiac repair by the macrophage recruitment and the production of pro‐inflammatory cytokines and chemokines,^111^, ^112^ the sustained inflammation that is mainly controlled by cardiac‐infiltrated CD4^+^ T cells participates in the development of cardiac fibrosis and dysfunction.^113^ Notably, immune therapeutics inhibiting CD4^+^ T cells have been found to prevent fibrotic pathology and dysfunction in the ischemic heart.^114^ The detrimental impacts of cardiac–infiltrated CD4^+^ T cells have also been detected in pressure overload‐promoted cardiac fibrosis and hypertrophy, which was found to be reversed by genetic inactivation of CD4^+^ T cells.^115^ Altogether, such results emphasize the importance of cardiac activated CD4^+^ T cells in the abnormal cardiac remodelling.

Exosomes secreted from activated CD4^+^ T cells (T‐activated EXs) have been identified as the important mediators in the myofibroblast activation, which is the core element for cardiac fibrotic remodelling post‐MI.^116^ It was shown that T‐activated EXs containing miR‐142‐3p are highly uptaken by cardiac fibroblasts and thereby deteriorate cardiac fibrosis post‐MI.^116^ The exosomal miR‐142‐3p is found to directly target and down‐regulate the adenomatous polyposis coli (APC), which leads to induction of β‐catenin degradation in the cytoplasm, resulting in the inhibition of the canonical WNT signalling cascade and, thereby, triggering myofibroblast activation and fibrogenesis.^116^ The APC protein is a positive regulator for the glycogen synthase kinase‐β (GSK‐β), a key protein included in cardiac remodelling.^117^ Thus, the T‐activated EXs can confer the pro‐fibrotic effects through modulating APC‐GSK‐β‐β‐catenin signal cascade mediated by miR‐142‐3p. Besides, the myofibroblasts activated by T‐activated EXs‐miR‐142‐3p might release bioactive molecules, such as TGF,^118^, ^119^ to induce the hypertrophic response of cardiomyocytes, leading to pathological cardiac remodelling.^116^ Supporting is the in vivo study on the MI mice that revealed the intravenously injected T‐activated EXs containing miR‐142‐3p move into the heart and worsen cardiac dysfunction after MI, as detected by the larger left ventricular end‐systolic diameter (LVESD) and left ventricular end‐diastolic diameter (LVEDD), as well as the smaller ejection fraction (EF%) and fractional shortening (FS%). To sum up, T‐activated EXs are the critical signal carriers in cardiac fibrosis following MI, and exosomal miR‐142‐3p serves as the signal conductor, providing a potential therapeutic target for treating MI‐related cardiac fibrosis.^116^

## 3 CONCLUSION

**FIGURE 1:**
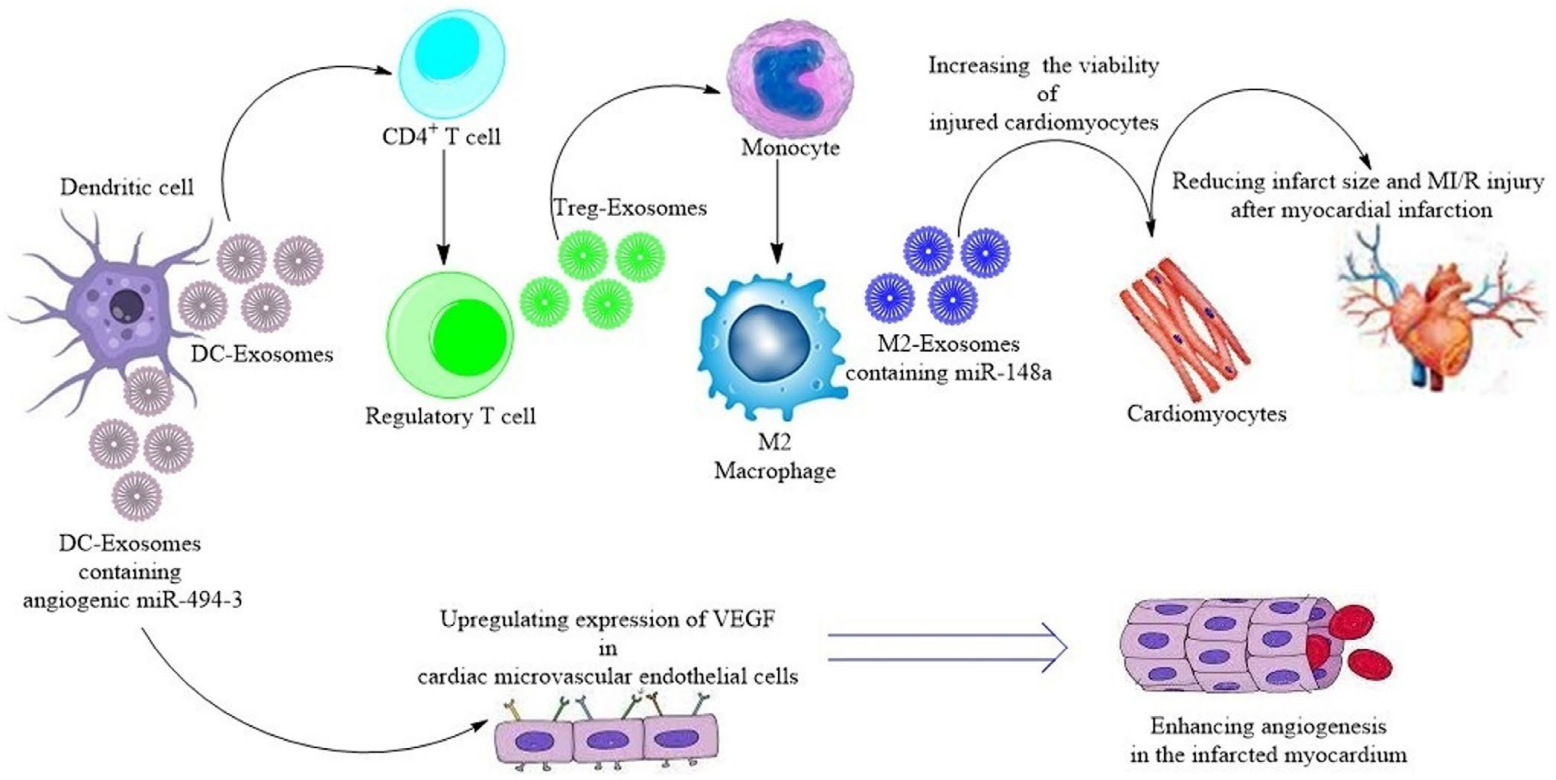
A schematic view representing exosome‐mediated intercellular crosstalk between cardioprotective immune cells and between immune cells with cardiac myocytes and endothelial cells

A balanced inflammatory process mediated by various immune cells and inflammatory factors has critical roles in the process of myocardial necrosis and repair following MI. Unrestrained inflammatory response or inappropriate inhibition of inflammation can influence the myocardial repair, resulting in ventricular remodelling as well as worsening cardiac dysfunction and progression of HF post‐MI. Thus, modulating the inflammation process after MI may provide a potential therapeutic approach. As crucial mediators of intercellular crosstalk, exosomes are effective in regulating immune cells and immune responses following MI, facilitating the reparative process of infarcted myocardium and preserving ventricular function via the communication between immune cells or between immune cells and cardiac cells (Table 1). DC‐EXs are proposed to activate CD4^+^ cells to cardioprotective anti‐inflammatory Tregs and, thereby, beneficially influence wound healing and survival. Following MI, Tregs infiltrate into infarcted myocardium where secrete exosomes that induce macrophage polarization towards M2 phenotype and promote a reduction in the pro‐inflammatory mediators and an increase in the anti‐inflammatory mediators, and thus suppress apoptosis of myocardial cells and decrease the myocardial infarct size, thereby ameliorating the cardiac damage and improving wound healing response and the fibrovascular scar formation. Besides, M2‐EXs transport high levels of miR‐148a from M2 macrophages into cardiomyocytes and, consequently, increase the viability of injured cardiomyocytes and cause a reduction in infarct size and MI/R injury. Moreover, miR‑494‑3‐enriched MI‐DC‐EXs could be directly taken up by the cardiac microvascular endothelial cells (CMECs), significantly up‐regulate the expression of the VEGF in CMECs, and thereby enhance angiogenesis in the infarcted myocardium after experimental MI (Figure 1). In contrast to the aforementioned helpful immune cell‐derived exosomes, there are detrimental exosomes secreted by pro‐inflammatory M1 macrophages and CD4^+^ T cells. Of note, M1‐EXs deliver pro‐inflammatory miR‐155 from M1 macrophages into endothelial cells, and thereby reducing the angiogenic ability of endothelial cells, exacerbating the myocardial injury and hindering cardiac healing. Also, T‐activated EXs can confer the pro‐fibrotic effects and induce the hypertrophic response of cardiomyocytes, leading to pathological cardiac remodelling and cardiac fibrosis post‐MI.

In conclusion, systemic deliveries of helpful exosomes or specific targeting of detrimental exosomes can provide an effective therapeutic window for treating MI‐injured myocardium. However, further experimental studies are necessary to understand the exact effects of these exosomes as well as other immune cells, like neutrophils, mast cells and B lymphocytes, in the infracted myocardium after MI.

## CONFLICT OF INTEREST

The authors declare that there are no conflicts of interest and financial support for the present review article.

## AUTHOR CONTRIBUTION

**Heling Wen:** Investigation (equal); Writing‐original draft (equal). **Lei Peng:** Project administration (equal); Writing‐original draft (equal). **Yu Chen:** Conceptualization (lead); Project administration (lead); Supervision (lead); Validation (lead).

## Data Availability

Data sharing not applicable to this article as no data sets were generated or analysed during the current study.
